# Age, atherosclerosis and type 2 diabetes reduce human mesenchymal stromal cell-mediated T-cell suppression

**DOI:** 10.1186/s13287-015-0127-9

**Published:** 2015-08-08

**Authors:** Ozge Kizilay Mancini, Dominique Shum-Tim, Ursula Stochaj, José A. Correa, Inés Colmegna

**Affiliations:** Department of Anatomy and Cell Biology, McGill University, Montreal, QC Canada; Division of Cardiothoracic Surgery and Surgical Research, Royal Victoria Hospital, McGill University Health Center, Montreal, QC Canada; Department of Physiology, McGill University, Montreal, QC Canada; Department of Mathematics and Statistics, McGill University, Montreal, QC Canada; Division of Rheumatology, Department of Medicine, McGill University, Montreal, QC Canada; Royal Victoria Hospital, McGill University Health Centre, 1001 Boulevard, Décarie, Montréal, Québec H4A 3J1 Canada

## Abstract

To this end human MSCs were isolated from adipose tissue and the MSC:CD4^+^ T-cell suppression was assessed in a co-culture system. In summary, this study demonstrates that advanced age, atherosclerosis and type 2 diabetes mellitus reduce the functional potency of MSCs. Optimizing the criteria for the selection of MSC donors could enhance the results of cell-based therapies.

**Electronic supplementary material:**

The online version of this article (doi:10.1186/s13287-015-0127-9) contains supplementary material, which is available to authorized users.

## Findings

Age and age- associated conditions (type 2 diabetes and athrosclerosis) decrease the immunomodulatory capacity of MSCs.

Optimal donor selection and proper immunological characterization of MSCs are critical for the successful therapeutic use of MSCs.

## Introduction

Atherosclerotic ischemic heart disease is the leading cause of death in developed countries. The prevalence, incidence, and severity of atherosclerosis (ATH) markedly increase with chronological age and in the context of age-associated chronic inflammatory conditions such as type 2 diabetes mellitus (T2DM) [1]. Chronic inflammation is a key regulatory process that links multiple risk factors for ATH and its complications to altered arterial biology. In mature atherosclerotic lesions, immune responses mediated by CD4^+^ T cells seem to be critical to accelerate atherogenesis and to promote plaque instability [2]. This is supported by the correlation between increased circulating numbers of activated CD4^+^ T cells and the extent of ATH in carotid and coronary arteries, and by the larger number of these cells in unstable plaques compared to those from patients with stable coronary artery disease [3, 4]. In vivo studies also showed the arrest in the development and progression of ATH following T cell-targeted therapy (i.e., anti-CD3Ab) [5]. The fundamental role of immune-activation in ATH provides the rationale to develop therapeutic interventions that restore immune homeostasis in ischemic heart disease. Among these strategies, the use of mesenchymal stromal cells (MSCs) showed promise in preclinical studies and most recently in patients with nonrevascularizable ischemic myocardium (reviewed in [6, 7]). Immunosuppressive and anti-inflammatory effects of MSCs are key mechanisms underlying their therapeutic effects [8]. A critical aspect linked to the success of any type of cell therapy is the appropriate selection of donors; however, the effect of donor’s age and age-associated co-morbidities on human MSC-mediated T-cell suppression remains undefined [9, 10]. The aim of this study was to evaluate the impact of chronological aging and of the age-associated diseases, ATH and T2DM, on the immunomodulatory capacity of MSCs.

## Methods

The McGill University Health Center Ethics Review Board approved the study and participants provided written informed consent. Subcutaneous adipose tissue was obtained from patients undergoing programmed cardiovascular surgery. Table 1 summarizes the demographics and cardiovascular risk factors of the studied subjects. A full description of methods is provided as supplementary data (Additional file 1: Supplementary materials and methods). Briefly, MSCs were derived from adipose tissue and proven to meet the International Society for Cellular Therapy definition criteria [11]. Freshly harvested, early passage (P4) MSCs were used in all assays. Peripheral blood mononuclear cells (PBMCs) were obtained from a single unrelated donor, monocyte depleted (<5 % monocytes) [12], Carboxyfluorescein succinimidyl ester (CFSE) stained and activated with CD3/CD28 beads. MSC-dependent CD4^+^ T-cell suppression was assessed in co-cultures [13]. Proliferation curves of live CD4^+^ T cells were plotted and the suppressive effect of MSCs on T cells was determined by comparing maximal proliferation (T cells alone) versus proliferation in co-cultures (MSCs + T cells) (Additional file 2: Figure S1). Wilcoxon's Rank Sum test was used for group comparisons. Multiple linear regression analysis examined the effects of age, ATH and T2DM on the mean MSC:CD4^+^ T-cell suppression capacity, after adjusting for the covariates of interest. Assumptions of the regression model were investigated with graphical analysis of residuals. All analyses were performed using SAS version 9.2 (SAS Institute Inc., Cary, NC, USA). All hypotheses tests were two-sided and performed at a significance level of 0.05.

**Table 1:** Demographic characteristics of the study subjects

	Surgical procedure	
	Coronary artery by-pass	Valve replacement
	graft (ATH)	(non-ATH)
	n = 41	n = 9
Sex (female:male, n)	13:28	5:4
Age in years, mean (SD)	63.4 (13.3)	59.7 (14.8)
Ethnicity (Caucasian:Asian, n)	37:4	9:0
Body mass index, mean (SD)	29.1 (9.2)	27.3 (12.1)
Cardiovascular risk factors, n (%)		
Tobacco	20 (49)	4 (44)
Hypertension	33 (80)	6 (66)
Hypercholesterolemia	33 (80)	4 (44)
Type 2 diabetes	12 (29)	4 (44)
Medications, n (%)		
Statins	33 (80)	5 (55)
ACE inhibitors/ARB	20 (49)	2 (22)
Beta blockers	26 (63)	2 (2)

*ACE* angiotensin-converting enzyme, *ARB* angiotensin II receptor blockers, *ATH* atherosclerosis

## Results

### MSCs from older donors are less efficient at suppressing T-cell proliferation

**Fig. 1:**
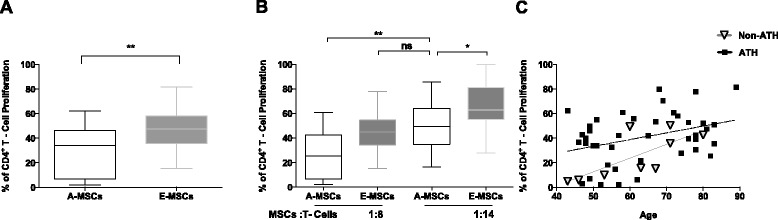
Age-associated decline in mesenchymal stromal cell (MSC)-mediated CD4^+^ T-cell suppression capacity. **a** MSCs from elderly donors (E-MSCs, ≥65 years, n = 23) are less efficient than those of non-elderly adults (A-MSCs, <65 years, n = 27) to suppress CD4^+^ T-cell proliferation at 1:8 MSC:CD4^+^ T cell ratio (**p = 0.003). **b** The suppressive effect of MSCs on CD4^+^ T cells depends on the MSC:CD4^+^ T-cell ratio (**p = 0.004). Twice the number of E-MSCs are required to affect CD4^+^ T-cell suppression to the same extent as A-MSCs (*p* > 0.9). **c** The effect of MSC donor age on the decline of CD4^+^ T-cell suppression is observed in patients with atherosclerosis (ATH; n = 18; *p* = 0.02, r = 0.4) and without ATH (non-ATH; n = 9; *p* = 0.02, r = 0.7)

The immunomodulatory capacity of adult MSCs (A-MSCs, <65 years old, n = 27) and elderly MSCs (E-MSCs, ≥65 years old, n = 23) was examined by analyzing their ability to inhibit the proliferation of anti-CD3/CD28-activated CD4^+^ T cells. The suppressive effect of A-MSCs and E-MSCs on CD4^+^ T-cell proliferation was dose-dependent. At a MSC:T cell ratio of 1:8 (Fig. 1a), A-MSCs (median 33.9 %, interquartile range (IQR) 6.8–46.0, n = 27) inhibited activated CD4^+^ T cells more effectively than E-MSCs (median 47.5 %, IQR 35.6–58.0, n = 23) (*p* < 0.003). Similar results were obtained at MSC:T cell ratios of 1:14 and 1:20. A-MSCs at 1:14 ratio (median 50.1 % IQR 37.6–62.2, n = 17) had similar inhibitory capacity as E-MSCs at 1:8 ratio, highlighting the magnitude of the E-MSC defect (Fig. 1b). MSC donor age positively correlated with T-cell proliferation in both ATH and non-ATH (samples from patients undergoing valve replacement surgery) groups (Pearson’s r = 0.4 and 0.7, respectively) (Fig. 1c), indicating an age-associated decline in the MSC immunomodulatory capacity. Similar defects of the E-MSC suppressive ability were observed on CD8^+^ T cells (Additional file 3: Figure S2). In a multiple linear regression model (Table 2), adjusting for covariates of interest, age had a significant effect on the reduction of MSC:CD4^+^ T-cell suppression (*p* = 0.02), with increasing mean CD4^+^ T-cell proliferation of 0.5 % (95 % confidence interval (CI) 0.1–1.0) for any 1-year increase in age.

**Table 2:** Results of linear regression analysis of factors associated with mesenchymal stromal cell immunomodulatory function

Variable	Estimate	Standard error	T	*p* value
Intercept	−5.62	16.28	−0.35	0.73
Presence of ATH	**21.61**	**7.68**	**2.82**	**0.01**
Presence of T2DM	**14.37**	**5.80**	**2.48**	**0.02**
Age	**0.54**	**0.23**	**2.42**	**0.02**
Sex	2.58	5.90	0.44	0.66
Tobacco	−4.55	5.72	−0.80	0.43
LVD	−1.67	6.76	−0.25	0.81
Statins	−9.00	6.96	−1.44	0.16
ACE inhibitors/ARB	–4.05	5.72	–0.71	0.48
Beta blockers	−5.14	6.42	−0.80	0.43

Entries in bold are statistically significant. *ACE* angiotensin-converting enzyme, *ARB* angiotensin II receptor blockers, *LVD* left ventricular dysfunction, *T2DM* type 2 diabetes mellitus

### ATH and T2DM reduce the immunomodulatory capacity of MSCs

**Fig. 2:**
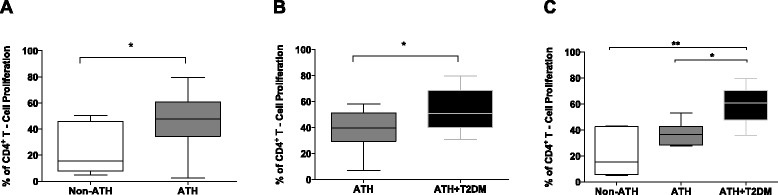
Reduced MSC-mediated T-cell suppression capacity in patients with atherosclerosis and type 2 diabetes. **a** MSCs from patients with atherosclerosis (ATH; n = 18; *p = 0.02) have a decreased capacity to suppress CD4^+^ T-cell proliferation at 1:8 MSC:CD4^+^ T cell ratio compared to age-matched controls without atherosclerosis (non-ATH, n = 9). **b** MSCs from patients with ATH (n = 12) and type 2 diabetes mellitus (T2DM) (n = 12) have impaired suppressive capacity compared to age-matched ATH controls (*p = 0.04). **c** MSC function is compromised in age-matched patients with chronic inflammatory diseases (non-ATH < ATH < ATH+T2DM; n = 7 per group; *p = 0.02, **p = 0.002)

The effect of biological aging on human MSC-mediated T-cell suppression was tested by evaluating MSCs from donors with ATH and T2DM, diseases associated with chronic inflammation and premature aging. MSCs from patients undergoing valve replacement surgery who had a normal pre-surgical coronary angiography (non-ATH subjects; median 15.6 %, IQR 9.9–42.5, n = 9) had a higher ability to suppress activated CD4^+^ T cells compared to MSCs from ATH age-matched controls (median 44.2, IQR 36.7–55.7, n = 18) (Fig. 2a). Moreover, in age-matched ATH patients the diagnosis of T2DM further reduced the MSC suppressive capacity (median 41.0, IQR 32.3–50.1, and 56.4, IQR 40.8–74.2, for non-T2DM/T2DM, n = 12, respectively) (Fig. 2b). Unadjusted, age-matched comparisons of MSC function in a sample subset (n = 7) suggested that ATH and T2DM were associated with a reduction in the MSC suppressive capacity (Fig. 2c). In the multiple regression model (Table 2), the presence/absence of ATH and T2DM in MSC donors had significant effects on their capacity to suppress proliferating CD4^+^ T cells. Subjects with ATH had a higher mean percentage of proliferating CD4^+^ T cells (decreased MSC:T-cell suppressive capacity) than those without ATH (mean difference 21.6 %; 95 % CI, 36.1–37.1). Similarly, subjects with T2DM had a higher mean percentage of proliferating CD4^+^ T-cells (mean difference 14.4 %; 95 % CI, 2.6–26.1). There was no statistically significant interaction either between ATH and T2DM or between each of them and age. This suggests that the effect of ATH is independent of T2DM and that these effects are also independent of age.

## Discussion

The PRECISE trial demonstrated the feasibility, safety and potential therapeutic benefit of the transendocardial administration of autologous adipose-derived MSCs in no-option patients with ischemic cardiomyopathy [7]. This study provided a proof of concept to test MSCs in larger ongoing clinical trials [6]. However, the improvement of cardiac function varied in preclinical trials. This emphasizes the need to define determinants of MSC therapeutic efficiency that will inform on proper donor selection [7, 14]. Previous data suggest that the aging process may impair the functional activity of murine MSCs, limiting their therapeutic potential [15]. Delineating the effects of aging and age-associated conditions on MSC function is critical, since the vast majority of patients that would benefit from MSC use in the context of ATH are elderly individuals. Notably, a significant proportion of this cohort also has T2DM [16]. Furthermore, defining the factors that impact MSC function would help to identify proper criteria to select the most therapeutically efficient cells for clinical applications.

Recent evidence confirmed that early-passage (i.e., passages 2–4) and freshly harvested MSCs have better in vitro T-cell suppression capacity and are associated with more clinical benefits than late-passage and freeze/thawed MSCs [17]. Our findings suggest that even when tested under “optimal” conditions (i.e., fresh early-passage MSCs in a reproducible immunopotency assay [13]), MSCs from elderly subjects with ATH have impaired T-cell suppression compared to their non-elderly adult counterparts. Consistent with previous studies [18, 19], this impaired function was not explained by differences in the MSC proliferative capacity or phenotype. Similar to E-MSCs, MSCs from donors with ATH and T2DM have reduced MSC-mediated T-cell suppression, and the coexistence of these chronic inflammatory conditions further compromise MSC function.

The overall efficacy of stem cell transplantation relies on the activity of donor cells and tissue environment. Our co-culture model is limited to MSCs and PBMCs and does not fully simulate all the components of the *in vivo* ischemic and inflammatory environment. However, this system predicts the MSC immunomodulatory potency, which is the most relevant mechanism for the therapeutic effect of MSCs [13]. The finding that age, T2DM and ATH are associated with reduced MSC immunomodulatory function is in line with a recent report suggesting that MSCs from subjects over 60 years of age have a reduced ability to ameliorate myocardial function compared to patients younger than 40 years [20]. A reduction of MSC-secreted angiogenic factors [18], an increased vulnerability to hypoxic injury [21] and higher levels of miR-335 are mechanisms proposed to account for the diminished reparative activities of E-MSCs.

## Conclusions

In summary, our results indicate that age and age-associated conditions (T2DM and ATH) decrease the immunomodulatory capacity of MSCs, highlighting the relevance of donor selection and the need for proper immunological characterization of MSCs. Understanding the interplay between aging, MSC function and their clinical implications remains the only rational path to the successful therapeutic use of MSCs.

## Additional files

Additional file 1: **Supplementary materials and methods.** (DOCX 46 kb)

Additional file 2: Figure S1. Gating strategy for the MSC:T cell suppression assay. The capacity of MSCs to suppress proliferative responses on activated CD4^+^ T cells was assessed in a 4-day allogeneic co-culture system. (A) PBMCs expanded for 4 days were used as controls (‘maximal proliferation’). (B) MSCs from different donors were co-cultured for 4 days with primary monocyte-depleted PBMCs obtained from a single unrelated donor. At day 4, PBMCs were stained with 7-aminoactinomycin D (7-AAD), and CD4-APC and flow cytometry was performed. The expansion index of 7AAD^−^CD4^+^ T cells was calculated with FlowJo. The percentage of CD4^+^ T-cell proliferation was calculated according to the following formula: % of Proliferation = (X – Control) / (Maximal Proliferation – Control) × 100, where X = Expansion index of MSC-CD4^+^ T cell co-culture for each sample, Control = Expansion index of non-stimulated CD4^+^ T cells, and Maximal Proliferation = Expansion index of CD4^+^ T cells stimulated with anti-CD3/CD28 beads in the absence of MSCs. (PPT 538 kb)

Additional file 3: Figure S2. Age-associated reduction in MSC-mediated CD8^+^ T-cell suppression. (A) The capacity of MSCs from non-elderly adult donors (A-MSCs, <65 years, n = 8) to suppress CD8+ T cells is higher than that of elderly donors (E-MSCs, ≥65 years, n = 8) (*p = 0.04; MSC:CD8^+^ T cell ratio of 1:8). (B) The E-MSC:CD8^+^ T-cell suppressive potency at 1:8 ratio is similar to that of A-MSCs at 1:14 ratio. (C) Age-dependent decline in MSC:CD8^+^ T-cell suppression in ATH patients (*p* = 0.01, R^2^ = 0.35). (D) Correlation of the suppressive effect of MSCs on CD4^+^ and CD8^+^ T cells (*p* < 0.0001, R^2^ = 0.7; MSC:T cell ratio of 1:8). (PPT 142 kb)

## Abbreviations

A-MSC: Adult mesenchymal stromal cell
ATH: Atherosclerosis
CI: Confidence interval
E-MSC: Elderly mesenchymal stromal cell
IQR: Interquartile range
MSC: Mesenchymal stromal cell
non-ATH: Non-atherosclerosis (samples from patients undergoing valve replacement surgery)
PBMC: Peripheral blood mononuclear cell
T2DM: Type 2 diabetes mellitus
