# Erratum to: Age, atherosclerosis and type 2 diabetes reduce human mesenchymal stromal cell-mediated T-cell suppression

**DOI:** 10.1186/s13287-017-0504-7

**Published:** 2017-02-14

**Authors:** Ozge Kizilay Mancini, Dominique Shum-Tim, Ursula Stochaj, José A. Correa, Inés Colmegna

**Affiliations:** 1 Department of Anatomy and Cell Biology, McGill University, Montreal, QC Canada; 2 Division of Cardiothoracic Surgery and Surgical Research, Royal Victoria Hospital, McGill University Health Center, Montreal, QC Canada; 3 Department of Physiology, McGill University, Montreal, QC Canada; 4 Department of Mathematics and Statistics, McGill University, Montreal, QC Canada; 5 Division of Rheumatology, Department of Medicine, McGill University, Montreal, QC Canada; 6 Royal Victoria Hospital, McGill University Health Centre, 1001 Boulevard, Décarie, Montréal, Québec H4A 3J1 Canada

## Erratum

The original article [1] contains a mistakenly shortened abstract due to a misinterpretation during its production.

As such, the full abstract should read as follows:

## Abstract

Atherosclerosis is an age-associated, multifactorial process driven by immune activation and inflammation. Ongoing clinical trials aim to establish the role of mesenchymal stromal cells (MSCs) as therapeutic agents in atherosclerosis. The beneficial effects of MSCs derive from their immune-modulatory properties. Understanding the impact of aging and age-associated conditions (i.e., type 2 diabetes mellitus and atherosclerosis) on MSC function is key to maximizing their therapeutic potency. The aim of this study was to assess the effect of chronological and biological aging on human MSC-mediated CD4+ T-cell suppression. To this end human MSCs were isolated from adipose tissue and the MSC:CD4+ T-cell suppression was assessed in a co-culture system. MSCs from elderly donors (≥65 years) had significantly lower T-cell suppressive capacity compared to those from donors <65 years (*p* = 0.003). Furthermore, MSCs from patients with atherosclerosis and type 2 diabetes mellitus were less efficient at suppressing T-cell proliferation (atherosclerosis, *p* = 0.02; type 2 diabetes mellitus, *p* = 0.04; compared to non-disease controls). Sex and tobacco use did not impact the immunosuppressive capacity of MSCs. In summary, this study demonstrates that advanced age, atherosclerosis and type 2 diabetes mellitus reduce the functional potency of MSCs. Optimizing the criteria for the selection of MSC donors could enhance the results of cell-based therapies.
