# Supplementary material for: Age, atherosclerosis and type 2 diabetes reduce human mesenchymal stromal cell-mediated T-cell suppression
Source: Stem Cell Res Ther. 2015 Aug 8;6(1):140. doi: 10.1186/s13287-015-0127-9 (PMC4529693; doi:10.1186/s13287-015-0127-9)
Supplement: Supplementary file 1 — Supplementary materials and methods. (DOCX 46 kb) [file 13287_2015_127_MOESM1_ESM.docx]

**DETAILED MATERIAL AND METHODS**

**Study subjects**

The McGill University Health Center Ethics Review Board approved the study and participants provided written informed consent. Subcutaneous adipose tissue was obtained from consecutive patients undergoing elective cardiovascular surgery (n=50). Samples from patients with atherosclerosis (ATH) requiring coronary artery bypass surgery (CABG) (n=41) were compared to those from patients without ATH (normal coronary angiogram) undergoing aortic valve replacement (n=9). Exclusion criteria for both groups were a history of systemic autoimmune diseases, cancer and acute or chronic infections. The demographic characteristics and cardiovascular risk factors of the patients included in the study are summarized in Table 1.

**Isolation of Multipotent Mesenchymal Stromal Cells**

Subcutaneous adipose tissue (1-4g) was washed extensively with phosphate-buffered saline (PBS), minced with surgical scissors and digested with 0.05% collagenase (Sigma-Aldrich Corporation, Missouri 63103 USA) dissolved in Hank's balanced salt solution (Invitrogen). Following the neutralization of the enzyme, the sample was centrifuged at 834 g for 5 min and the supernatant was discarded. The pellet was resuspended in complete medium (CM) (1.0g/L glucose, with L-glutamine & sodium pyruvate Dulbecco’s modified Eagle’s medium (DMEM) (Wisent Biotechnologies), supplemented with 10% MSC Qualified Fetal Bovine serum (FBS) 1% penicillin / streptomycin (10,000 unit / mL Penicillin, 10,000mg/mL Streptomycin - Life technologies). The cells were cultured under standard conditions (5% carbon dioxide; 37°C) in 75-cm^2^ tissue culture flasks (1gr of tissue/flask). Two days after isolation, non-adherent cells were washed off and CM was added. Subsequently, at 80% confluency mesenchymal stem cells (MSCs) were trypsinized and subcultured at a density of 5000 cells / cm^2^ [1] .

**Flow cytometric detection of cell surface receptors**

Immunophenotypic characterization of MSCs was performed according to criteria established by the International Society for Cellular Therapy [2] by multiparametric flow cytometry (BD LSRII; Becton Dickinson Co, Mountain View, CA). Passage 2 MSCs were treated with Fc receptor blocking reagent and stained with the following flurochrome-conjugated monoclonal antibodies (BD Biosciences): fluorescein isothiocyanate (FITC)-conjugated anti-CD90 and anti-CD45; phycoerythrin (PE)-conjugated anti-CD73; allophycocyanin (APC)-conjugated anti-CD34, anti-CD19 and anti-HLA-DR; peridinin chlorophyll (PerCP)-conjugated anti-CD105, anti-CD44 and anti-CD14]. Nonspecific staining was determined by incubation of similar cell aliquots with isotype controls. Data was analyzed with FlowJo software 9.7.2.

**Multilineage differentiation assays**

Passage 3 MSCs were plated in 24-well plates at a density of 5000 cells/cm^2^. At ~90% confluence, cells were incubated in one of the three differentiation mediums for 3 weeks as per manufactures protocol (StemPro® Adipogenesis, osteogenesis, chondrogenesis Differentiation Kit). Cells were then fixed with 4% formaldehyde and stained with alizarin red S (Sigma-Aldrich), oil red O (Sigma-Aldrich) or safranin O (Sigma-Aldrich) to assess osteogenic, adipogenic and chondrogenic differentiation, respectively.

**Peripheral blood mononuclear cells (PBMC) isolation, carboxyfluorescein succinimidyl ester (CFSE) fluorescent dye labeling, and PBMC stimulation**

PBMCs were freshly isolated for each experiment from the same donor (32 years old, healthy, female, no smoking). PBMCs were separated by Ficoll-Hypaque density gradient centrifugation (FICOLL 400*- Sigma-Aldrich) and cultured in 10 % FBS RPMI (Wisent Biotechnologies) medium overnight to deplete monocytes. The efficacy of monocyte depletion (95%) was verified by flow cytometry [3]. Monocyte-depleted PBMCs were stained with carboxyfluoroscein succinimidyl ester (CFSE) (Sigma) and stimulated with anti- CD3/CD28 beads (1 bead/cell) (Dynabeads® Human T-Activator CD3/CD28, life technologies) [4] [5].

**Co-cultures**

The capacity of MSCs to suppress proliferative responses on activated CD4^+^T cells was assessed in a 4-day allogeneic co-culture system (MSCs from different donors exposed to the same unrelated primary monocyte depleted PBMCs) [6]. MSCs were plated at 25 × 10^3^, 14 × 10^3^, 10× 10^3^, cells per well in flat-bottomed 96-well plates (Corning) and cultured overnight. Following activation 2 × 10^5^ monocyte depleted CFSE stained PBMCs were plated on MSCs (MSCs:PBMCs 1:8, 1:14, 1:20). PBMCs expanded for 4 days were used as controls (‘maximal proliferation’). At day 4 cells were stained with 7-Aminoactinomycin D (7-AAD), and CD4-APC. The Expansion Index of 7AAD-CD4+ cells was determined (FlowJo) and the percentage of CD4^+^ T-cell proliferation was calculated according to the following formula: % of Proliferation = X-Control/Maximal Proliferation-Control x 100 where X = Expansion index of MSC- CD4^+^ T-cells co-culture for each sample, Control= Expansion index of CD4^+^ T-cells without anti CD3/CD28 stimulation, and Maximal Proliferation = Expansion index of CD4^+^ T cells stimulated by anti-CD3/CD28 beads in the absence of MSC.

**Statistical Analysis**

Descriptive statistics summarize all study variables. For categorical variables we report counts and percentages whereas for continuous variables we report means and standard deviations when the distribution of values is normal; otherwise we report medians, and inter-quartile range.

Multiple linear regression analysis was used to investigate the effect of age and premature aging-associated conditions (atherosclerosis-ATS and diabetes mellitus-T2DM) on the mean MSC:T-cell suppression capacity. The regression model included covariates reported to potentially influence the outcome: sex, tobacco and treatment (use of statins, angiotensin-converting-enzyme inhibitors ± angiotensin II receptor blockers and/or β-blockers). Age was modeled as a continuous variable whereas ATH, T2DM and other covariates of interest were modeled as binary variables (presence/absence). Interactions between age, ATH, T2DM and the covariates of interest were assessed. Assumptions of the regression model (randomness of errors, homogeneity of variance, normality, presence of outliers) were investigated with a graphical analysis of residuals. All hypotheses tests were 2-sided and performed at a significance level of 0.05. All analyses were performed using SAS version 9.3 (SAS Institute, Inc. Cary NC, USA).

**References**

1. Bernacki SH, Wall ME, Loboa EG. Isolation of human mesenchymal stem cells from bone and adipose tissue. Methods in cell biology. 2008;86:257-78. doi:10.1016/S0091-679X(08)00011-3.

2. Dominici M, Le Blanc K, Mueller I, Slaper-Cortenbach I, Marini F, Krause D et al. Minimal criteria for defining multipotent mesenchymal stromal cells. The International Society for Cellular Therapy position statement. Cytotherapy. 2006;8(4):315-7. doi:10.1080/14653240600855905.

3. Barrett L, Dai C, Gamberg J, Gallant M, Grant M. Circulating CD14-CD36+ peripheral blood mononuclear cells constitutively produce interleukin-10. Journal of leukocyte biology. 2007;82(1):152-60. doi:10.1189/jlb.0806521.

4. Trickett A, Kwan YL. T cell stimulation and expansion using anti-CD3/CD28 beads. Journal of immunological methods. 2003;275(1-2):251-5.

5. Bloom DD, Centanni JM, Bhatia N, Emler CA, Drier D, Leverson GE et al. A reproducible immunopotency assay to measure mesenchymal stromal cell-mediated T-cell suppression. Cytotherapy. 2015;17(2):140-51. doi:10.1016/j.jcyt.2014.10.002.

6. Kronsteiner B, Wolbank S, Peterbauer A, Hackl C, Redl H, van Griensven M et al. Human mesenchymal stem cells from adipose tissue and amnion influence T-cells depending on stimulation method and presence of other immune cells. Stem cells and development. 2011;20(12):2115-26. doi:10.1089/scd.2011.0031.
