# Supplementary material for: Age, atherosclerosis and type 2 diabetes reduce human mesenchymal stromal cell-mediated T-cell suppression
Source: Stem Cell Res Ther. 2015 Aug 8;6(1):140. doi: 10.1186/s13287-015-0127-9 (PMC4529693; doi:10.1186/s13287-015-0127-9)
Supplement: Supplementary file 2 — Gating strategy for the MSC:T cell suppression assay. The capacity of MSCs to suppress proliferative responses on activated CD4+ T cells was assessed in a 4-day allogeneic co-culture system. (A) PBMCs expanded for 4 days were used as controls (‘maximal proliferation’). (B) MSCs from different donors were co-cultured for 4 days with primary monocyte-depleted PBMCs obtained from a single unrelated donor. At day 4, PBMCs were stained with 7-aminoactinomycin D (7-AAD), and CD4-APC and flow cytometry was performed. The expansion index of 7AAD−CD4+ T cells was calculated with FlowJo. The percentage of CD4+ T-cell proliferation was calculated according to the following formula: % of Proliferation = (X – Control) / (Maximal Proliferation – Control) × 100, where X = Expansion index of MSC-CD4+ T cell co-culture for each sample, Control = Expansion index of non-stimulated CD4+ T cells, and Maximal Proliferation = Expansion index of CD4+ T cells stimulated with anti-CD3/CD28 beads in the absence of MSCs. (PPT 538 kb) [file 13287_2015_127_MOESM2_ESM.ppt]

## Slide 1
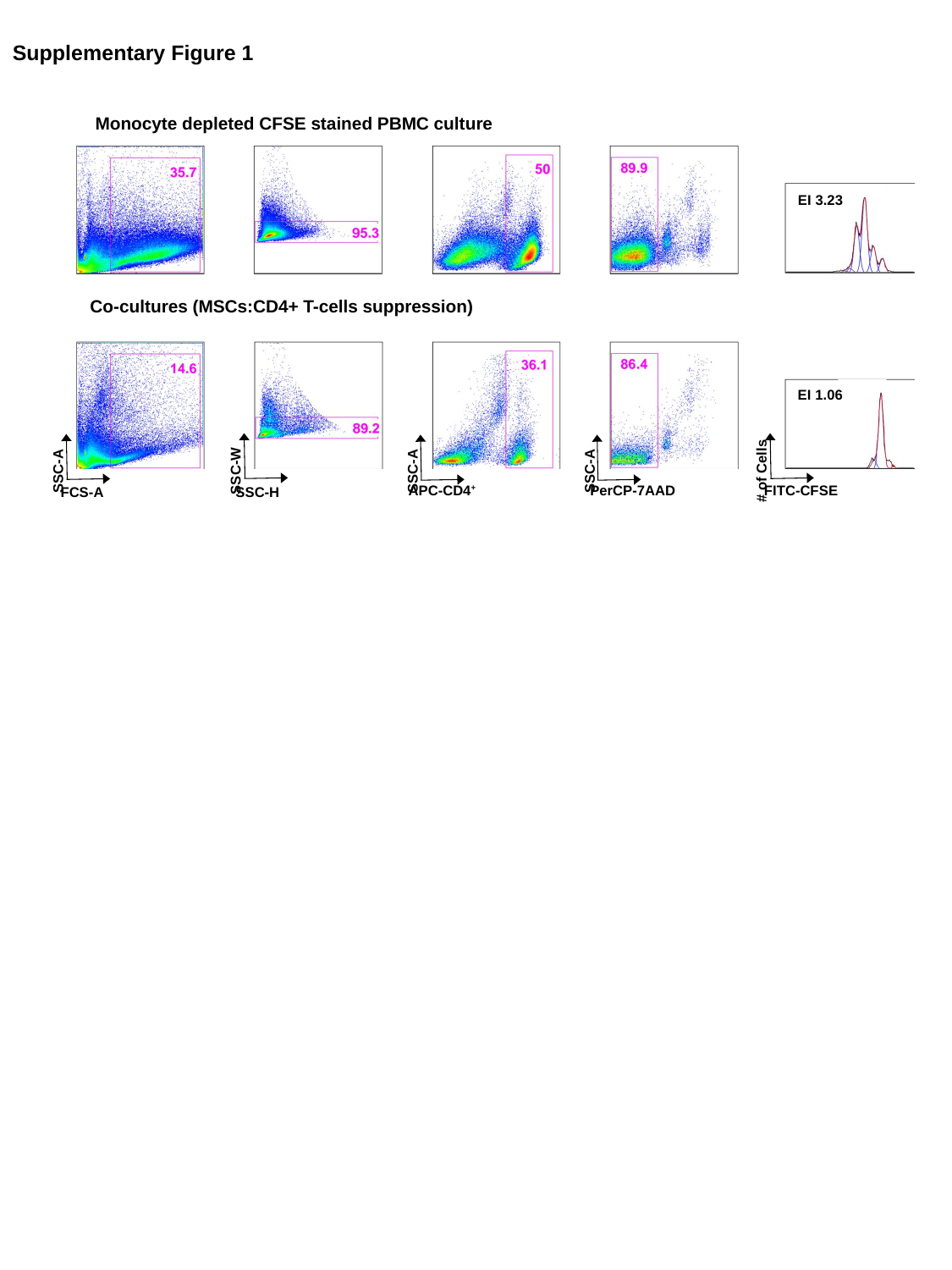

Supplementary Figure 1
Monocyte depleted CFSE stained PBMC culture
EI 3.23
Co-cultures (MSCs:CD4+ T-cells suppression)
EI 1.06
# of Cells
FITC-CFSE
SSC-W
SSC-H
SSC-A
FCS-A
SSC-A
APC-CD4+
SSC-A
PerCP-7AAD
