# Supplementary figures and images for: Age, atherosclerosis and type 2 diabetes reduce human mesenchymal stromal cell-mediated T-cell suppression
Source: Stem Cell Res Ther. 2015 Aug 8;6(1):140. doi: 10.1186/s13287-015-0127-9 (PMC4529693; doi:10.1186/s13287-015-0127-9)

## Slide 1
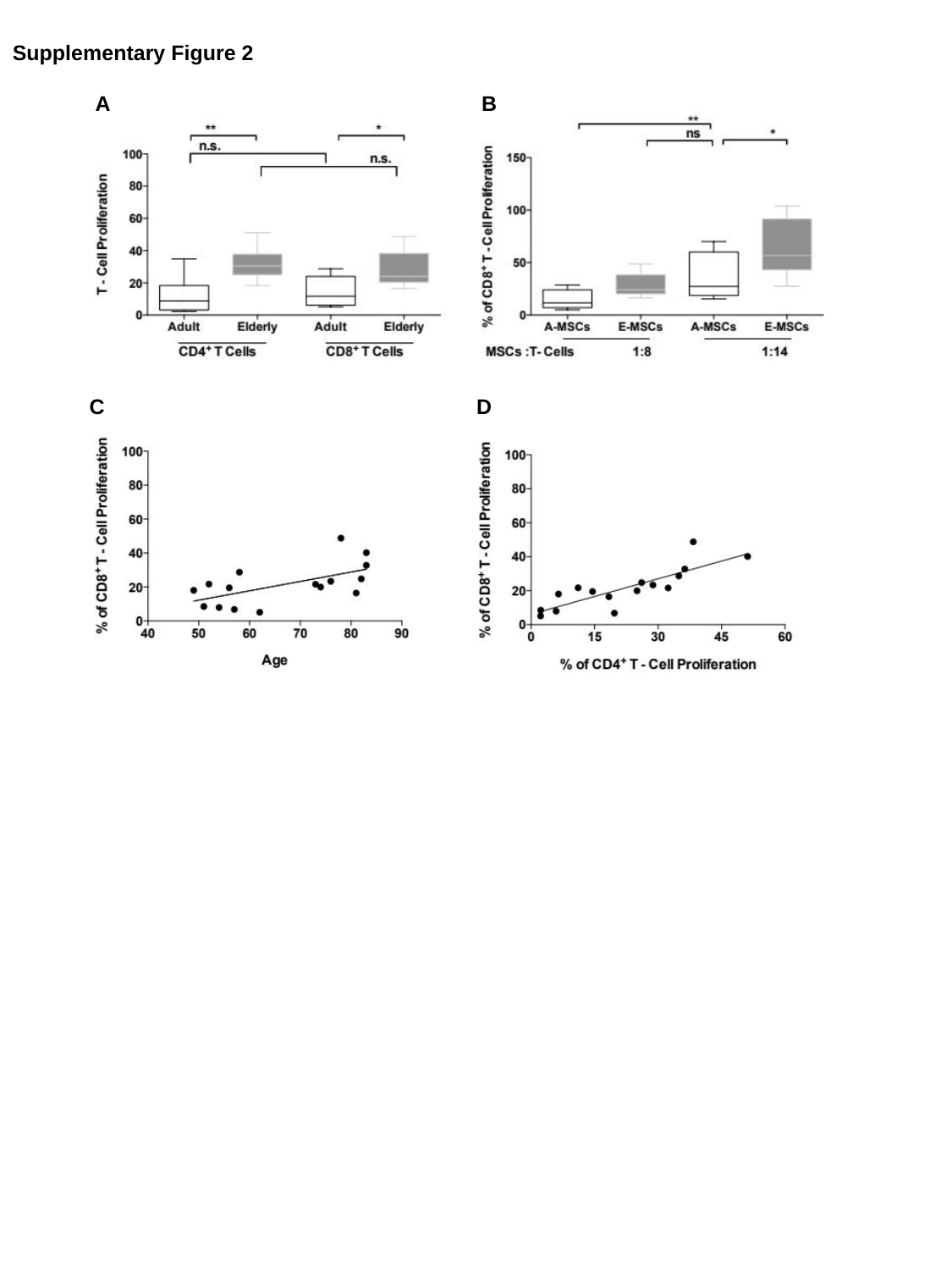

Supplementary Figure 2
 A B
 C D

Supplement: Supplementary file 3 — Age-associated reduction in MSC-mediated CD8+ T-cell suppression. (A) The capacity of MSCs from non-elderly adult donors (A-MSCs, <65 years, n = 8) to suppress CD8+ T cells is higher than that of elderly donors (E-MSCs, ≥65 years, n = 8) (*p = 0.04; MSC:CD8+ T cell ratio of 1:8). (B) The E-MSC:CD8+ T-cell suppressive potency at 1:8 ratio is similar to that of A-MSCs at 1:14 ratio. (C) Age-dependent decline in MSC:CD8+ T-cell suppression in ATH patients (p = 0.01, R2 = 0.35). (D) Correlation of the suppressive effect of MSCs on CD4+ and CD8+ T cells (p < 0.0001, R2 = 0.7; MSC:T cell ratio of 1:8). (PPT 142 kb) [file 13287_2015_127_MOESM3_ESM.ppt]
